# Oat (*Avena sativa*) Extract against Oxidative Stress-Induced Apoptosis in Human Keratinocytes

**DOI:** 10.3390/molecules26185564

**Published:** 2021-09-13

**Authors:** Sooji Song, Yoon-Mi Lee, Yu Young Lee, Kyung-Jin Yeum

**Affiliations:** 1 Department of Integrated Biosicences, College of Biomedical and Health Science, Konkuk University, Chungju-si 27478, Chungcheongbuk-do, Korea; ssj4037@naver.com (S.S.); yoonmilee@kku.ac.kr (Y.-M.L.); 2 Department of Biomedical Chemistry, College of Biomedical and Health Science, Konkuk University, Chungju-si 27478, Korea; 3 Department of Central Area, National Institute of Crop Science, Rural Development Administration, Suwon 16429, Korea; leeyy260@korea.kr

**Keywords:** oxidative stress, oat extract, DNA damage, H2AX, apoptosis, check-point kinase, caspase

## Abstract

Oat (*Avena sativa*) is well known for its various health benefits. The protective effect of oat extract against oxidative stress-induced apoptosis in human keratinocytes HaCaT was determined. First, extracts of two varieties of oat, Daeyang and Choyang, were analyzed for fat-soluble antioxidants such as α-tocotrienol, γ-oryzanols, lutein and zeaxanthin using an UPLC system and for antioxidant activity using a DPPH assay. Specifically, an 80% ethanol extract of Daeyang oat (*Avena sativa* cv. Daeyang), which had high amounts of antioxidants and potent radical scavenging activity, was further evaluated for protective effect against oxidative stress-induced cell death, intracellular reactive oxygen species levels, the phosphorylation of DNA damage mediating genes such as H2AX, checkpoint kinase 1 and 2, and p53 and the activation of apoptotic genes such as cleaved caspase-3 and 7 and poly (ADP-ribose) polymerase in HaCaT cells. The Daeyang and Choyang oat 80% ethanol extracts had 26.9 and 24.1 mg/100 g γ-oryzanols, 7.69 and 8.38 mg/100 g α-tocotrienol, 1.25 and 0.34 mg/100 g of lutein and 1.20 and 0.17 mg/100 g of zeaxanthin, respectively. The oat 80% ethanol extract treatment (*Avena sativa* cv. Daeyang) had a protective effect on oxidative stress-induced cell death in HaCaT cells. In addition, the oat 80% ethanol extracts led to a significant decrease in the intracellular ROS level at a concentration of 50–200 μg/mL, the attenuation of DNA damage mediating genes and the inhibition of apoptotic caspase activities in a dose dependent manner (50–200 μg/mL). Thus, the current study indicates that an oat (*Avena sativa* cv. Daeyang) extract rich in antioxidants, such as polyphenols, avenanthramides, γ-oryzanols, tocotrienols and carotenoids, has a protective role against oxidative stress-induced keratinocyte injuries and that oat may a useful source for oxidative stress-associated skin damage.

## 1. Introduction

It is well known that an accumulation of reactive oxygen species (ROS) such as hydrogen peroxide, superoxide anion, peroxynitrite and peroxyl radicals [1] can cause oxidative stress, damaging DNA and other biomolecules, leading to inflammation, aging and cancer [2,3,4,5]. The epidermis, the outermost of the three layers of the skin [6], is directly exposed to external stimuli and is vulnerable to oxidative stress. Considering that oxidative damage to the epidermis can trigger the pathogenesis of the skin [7,8], protecting skin from oxidative stress can be pivotal to the body’s defense system. Therefore, scientists have paid attention to bioactive substances that can alleviate skin damage by protecting against oxidative stress [9,10,11].

The consumption of whole grain oats (*Avena sativa*) is well known for its positive health benefits against metabolic disorders such as hypercholesterolemia, high blood pressure and hyperglycemia [12,13]. These beneficial effects of oats can be largely explained by the role of their antioxidative compounds such as tocotrienols, flavonoids [14], phenolics [15,16] and avenanthramides [17], and soluble fibers such as beta-glucan [12]. More recently, oat extracts such as oat oil [18,19] as well as oat plantlet extracts [20,21,22,23,24] have been studied for their anti-inflammatory and skin barrier functions. However, the molecular mechanisms of oat extract on the anti-apoptotic properties (key function in regulating skin development) remains unknown.

Therefore, in this study, the protective effect of an oat (*Avena sativa* cv. Daeyang) extract rich in antioxidants against cellular damage caused by oxidative stress such as hydrogen peroxide and UVB was investigated using human keratinocytes, a major cell type of the epidermis.

## 2. Results and Discussion

### 2.1. Bioactive Components of Oat (Avena sativa) Extracts

Fat-soluble bioactive components in the 80% ethanol extract of oat (*Avena sativa* cv. Daeyang and Choyang) were analyzed. Daeyang and Choyang oats had 26.9 and 24.1 mg/100 g of γ-oryzanols, 7.69 and 8.38 mg/100 g of α-tocotrienol, 1.25 and 0.34 mg/100 g of lutein and 1.20 and 0.17 mg/100 g of zeaxanthin, respectively. Bioactive components such as γ-oryzanols [25,26], α-tocotrienol [27], xanthophylls [28] and avenanthramide [29] in oats are well known for their antioxidant activities. This study confirmed the abundance of bioactive substances in oats, and the Daeyang cultivar had higher amounts of fat-soluble antioxidants than the Choyang oats. We believe these fat-soluble as well as water-soluble bioactive substances in oat synergistically interact, as previously reported in other plant bioactives in vitro [30,31,32] and in vivo [33], to exert antioxidant functions in the biological system.

### 2.2. Antioxidant Activity of Oat (Avena sativa) Extract

**Figure 1:**
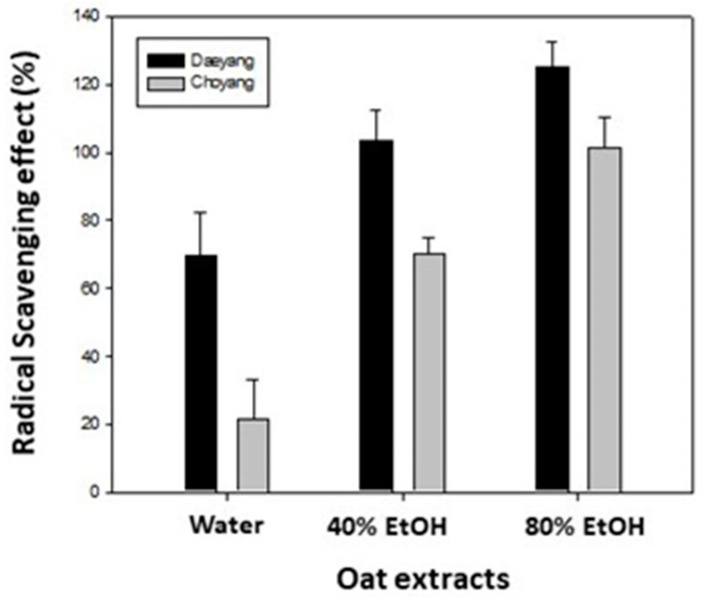
Antioxidant activities of oat extracts. In vitro 2,2-Diphenyl-1-picryl-hydrazyl-hydrate (DPPH) free radical scavenging activities of Daeyang and Choyang oat extracts at concentration of 5 mg/mL. Data are expressed as mean ± SD.

The free radical scavenging activity of oat extract was determined using a 2,2-diphenyl-1-picryl-hydrazyl-hydrate (DPPH) assay. Phenolic components in plant foods such as barley [34] and *Leonurus cardiaca* [35] are reported to be correlated with their radical scavenging activities. The DPPH radical scavenging activities of water, the 40% ethanol and the 80% ethanol extracts of Daeyang and Choyang cultivars are presented in Figure 1. The Daeyang extract had higher antioxidant activity than the Choyang extract for both the water and ethanol extracts, and the ethanol extracts had higher antioxidant activity than the water extract for both cultivars. In particular, the 80% ethanol extract of Daeyang cultivar had the highest DPPH radical scavenging activity (Figure 1). The Daeyang oats, which contained more fat-soluble antioxidants than the Choyang oats, also had higher radical scavenging activity. Therefore, the current study focused on the protective effect of an 80% ethanol extract of Daeyang oats (*Avena sativa* cv. Daeyang) against the cellular damage caused by oxidative stress using human keratinocytes HaCaT.

### 2.3. Protective Effect of Oat (Avena sativa cv. Daeyang.) Extract on Oxidative Stress-Induced Cell Death and Intracellular Reactive Oxygen Species Levels

**Figure 2:**
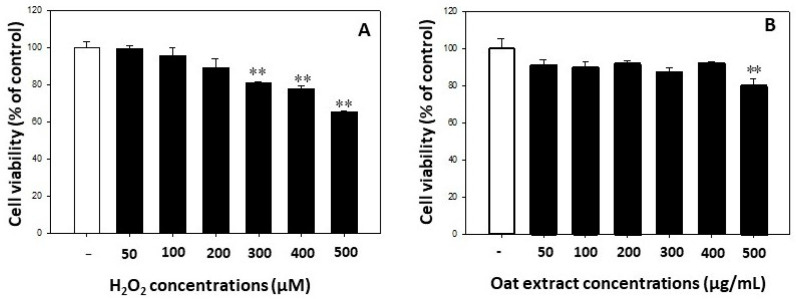
Effect of hydrogen peroxide (**A**) and oat 80% ethanol extract (**B**) on cell viabilities. Human keratinocyte HeCaT cells were treated with hydrogen peroxide or oat (*Avena sativa,* cv. Daeyang) 80% ethanol extract for 24 h followed by MTT assay to determine cell viabilities. Data are expressed as mean ± SD. ** *p* < 0.01 vs. vehicle-treated cells.

The exposure of human keratinocytes to H_2_O_2_ at concentrations of 50–200 μM for 24 h had no significant effect on cell viabilities. On the other hand, cell death was significantly increased at a H_2_O_2_ concentration of 300 μM or higher, and the viability reached 65% at a H_2_O_2_ concentration of 500 μM, as presented in Figure 2A. The oat 80% ethanol extract had no cytotoxicity in the dose range of 50–400 μg/mL to the keratinocytes, whereas a treatment of 500 μg/mL of oat extract resulted in significant cell death, as shown in Figure 2B. Therefore, 50–200 μg/mL of oat extracts and 500 μM of hydrogen peroxide were used for evaluating the protective effect of the oat 80% ethanol extract on the oxidative stress in keratinocytes.

**Figure 3:**
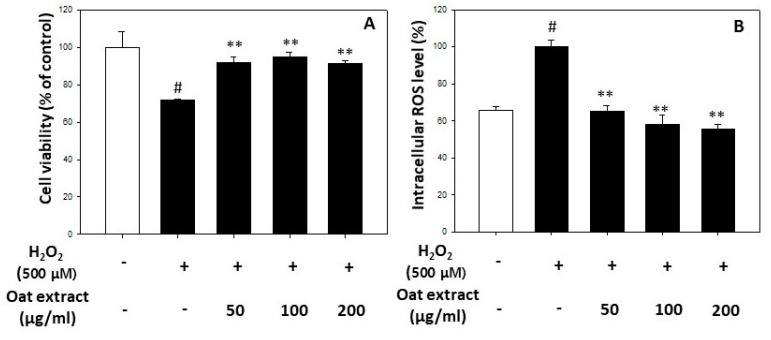
Protective effect of oat (*Avena sativa* cv. Daeyang) 80% ethanol extract on hydrogen peroxide-induced cell death (**A**) and intracellular reactive oxygen species (ROS) levels (**B**). To determine the cell viabilities, HaCaT cells were pre-treated with various concentrations of oat 80% ethanol extract for 24 h, then cell medium containing oat 80% ethanol extract was removed, and further incubated with hydrogen peroxide for 24 h followed by MTT assay. To determine the intracellular ROS levels, HaCaT cells were treated with various concentrations of oat 80% ethanol extract in the presence of hydrogen peroxide for 3 h followed by DCF-DA fluorescence assay. Data are expressed as mean ± SD. # *p* < 0.05 vs. vehicle-treated cells; ** *p* < 0.01 vs. H_2_O_2_ treated cells.

The pre-treatment of keratinocytes with the oat (*Avena sativa* cv. Daeyang) 80% ethanol extract significantly protected cell death induced by hydrogen peroxide, as presented in Figure 3A. It was found that the oat 80% ethanol extract at a concentration of 50 μg/mL was sufficient to maintain cell viability against oxidative stress-induced cell damage. We further determined whether the oat (*Avena sativa* cv. Daeyang) 80% ethanol extract had intracellular reactive oxygen species (ROS) scavenging effects. The intracellular ROS induced by hydrogen peroxide was also restored to the level before hydrogen peroxide treatment by oat 80% ethanol extracts, as shown Figure 3B. The recovery of the intracellular ROS level induced by hydrogen peroxide to the level without hydrogen peroxide treatment was also sufficient with an oat 80% ethanol extract concentration of 50 μg/mL.

### 2.4. Protective Effect of Oat (Avena sativa cv. Daeyang) Extract on Oxidative Stress-Induced DNA Damage

**Figure 4:**
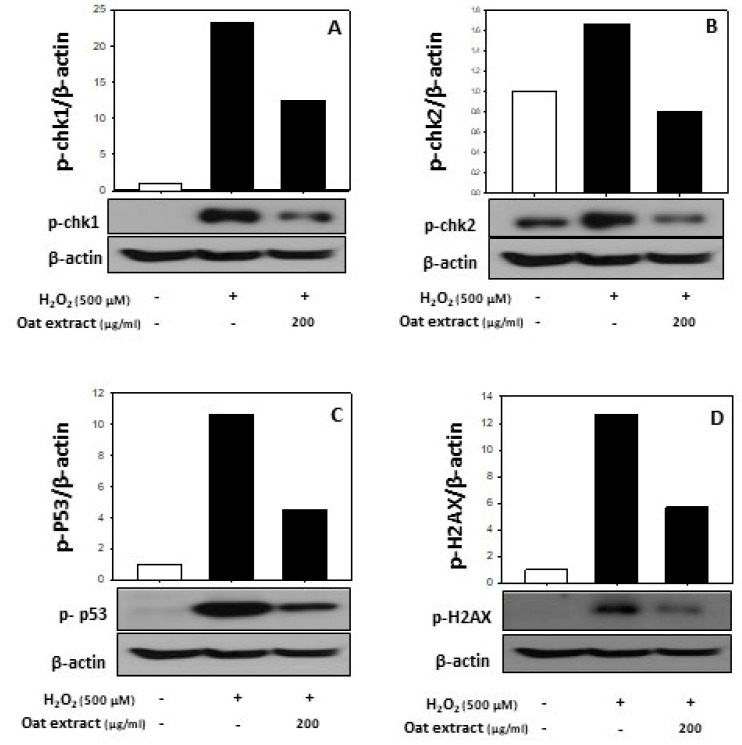
Effects of oat (*Avena sativa* cv. Daeyang) 80% ethanol extract on the expression of phospho-chk1 (**A**), phospho-chk2 (**B**), phospho-p53 (**C**) and phospho-H2AX (**D**) in human keratinocytes HaCaT. Cells were treated with various concentrations (50–200 μg/mL) of oat 80% ethanol extracts for 24 h, followed by treatment with 500 μM H_2_O_2_ for 2 h. Total cell lysates were prepared at the same time, and Chk-1, Chk-2, H2AX and p-53 protein levels were assessed using immunoblotting. β-Actin was used as an internal control.

The protective effect of the oat *(Avena sativa cv. Daeyang)* 80% ethanol extract against oxidative stress-induced DNA damage was determined on the expressions of sensor molecules that recognize DNA damage [36]. When checkpoint kinase 1 is activated, it phosphorylates a variety of substrate proteins leading to various cell responses such as the activation of DNA damage checkpoints, cell cycle arrest, DNA repair and apoptosis [37,38]. As presented in Figure 4, the hydrogen peroxide treatment was associated with a dramatic increase in the phosphorylation of DNA damage mediators, checkpoint kinase 1 (Chk1, Figure 4A) and checkpoint kinase 2 (Chk2, Figure 4B), while the oat 80% ethanol extract treatment led to significant decreases in phospho-chk1 and phospho-chk2 expressions in H_2_O_2_-treated HaCaT cells. Furthermore, the downstream target protein of Chk2, p53, reported to be medicated by various kinases in response to diverse stresses [39], was phosphorylated by hydrogen peroxide, while the activation was greatly reduced by the oat 80% ethanol extract treatment (Figure 4C). It is interesting to note that the oxidative stress-activated checkpoint kinases modulated the DNA damage response by activating the downstream target protein, p53 [40,41], and that the effect of the oat 80% ethanol extract on these DNA damage genes was consistent. It has been reported that the phosphorylation of the Ser-139 residue of the histone variant H2AX is an early cellular response to the induction of DNA double-strand breaks and this event is a highly specific and sensitive molecular marker for DNA damage [42]. In this study, an early sign of DNA damage, as determined by the phosphorylation of H2AX, was dramatically increased by hydrogen peroxide, whereas the elevated activation was dramatically reduced by the oat 80% ethanol extract treatment (Figure 4D).

**Figure 5:**
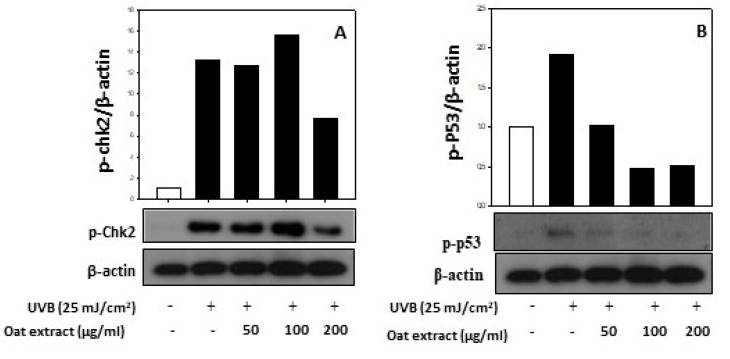
Effect of oat (*Avena sativa* cv. Daeyang) 80% ethanol extract on the expression of phospho-chk2 (**A**) and phospho-p53 (**B**) in human keratinocytes HaCaT. Cells were treated with various concentrations (50–200 μg/mL) of oat 80% ethanol extracts for 24 h, followed by incubation for 5 h after exposure of 25 mJ/cm^2^ of UVB. Chk-2 and p-53 protein levels were assessed using immunoblotting. β-Actin was used as an internal control.

As expected, the phosphorylation of checkpoint kinase 2 and p53 was also dramatically increased by the exposure of keratinocytes to UVB (25 mJ/cm^2^). In addition, the oat 80% ethanol extract treatment prevented the activation of these DNA damage-mediating genes by UVB, as presented in Figure 5. This protective effect of the oat 80% ethanol extract is in line with previous studies indicating the suppression of UVB-induced skin damage by natural products such as an algae-derived phenolic compound [43] and liquiritin [44].

### 2.5. Protective Effect of Oat (Avena sativa cv. Daeyang) Extract on Oxidative Stress-Induced Apoptosis

**Figure 6:**
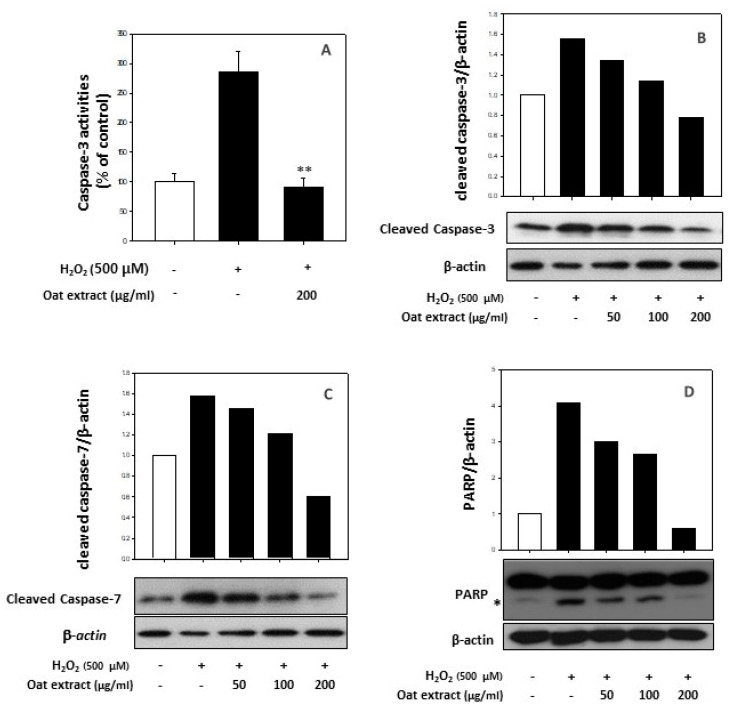
Effect of oat (*Avena sativa,* cv. Daeyang) 80% ethanol extract on caspase-3 enzyme activity (**A**), cleaved caspase-3 protein (**B**), cleaved caspase-7 protein (**C**) and cleaved PARP protein levels (**D**) in human keratinocytes HaCaT. The asterisk adjacent to the lower band of PARP indicates the cleaved PARP protein. Cells were treated with various concentrations (50–200 μg/mL) of oat 80% ethanol extracts for 24 h, followed by treatment with 500 μM H_2_O_2_ for 24 h. Caspase-3 enzymatic activity was determined using colorimetric assay. ** *p* < 0.01 vs. without oat extract. Cleaved caspase-3, cleaved caspase-7 and cleaved PARP protein levels were assessed using immunoblotting in the same sample set. β-Actin was used as a loading control.

To determine whether the oat 80% ethanol extract affected hydrogen peroxide-induced apoptotic cell death, several apoptotic markers have been evaluated. We determined caspases, which play a central role in the induction of apoptosis [45]. First, colorimetric analysis indicated that the oxidative stress led to an increase in caspase-3 enzymatic activities by 286.5% of the control value, whereas the oat (*Avena sativa,* cv. Daeyang) 80% ethanol extract treatment, at a concentration of 200 μg/mL, significantly reduced caspase-3 activity to 91.09 ± 15.08% of the control value, as presented in Figure 6A. Next, the modification of apoptotic genes by the oat 80% ethanol extract has been determined. The cleaved caspase-3 (Figure 6B) and cleaved caspase-7 (Figure 6C) protein levels elevated by hydrogen peroxide exposure were also dramatically reduced by the oat 80% ethanol extract treatment in a dose-dependent manner. Since the activation of poly (ADP-ribose) polymerase (PARP) is mediated by caspases and causes apoptosis [46,47], cleaved PARP has also been analyzed. As presented in Figure 6D, a treatment of the oat 80% ethanol extract recovers the cleaved PARP level in a dose-dependent manner as well.

**Figure 7:**
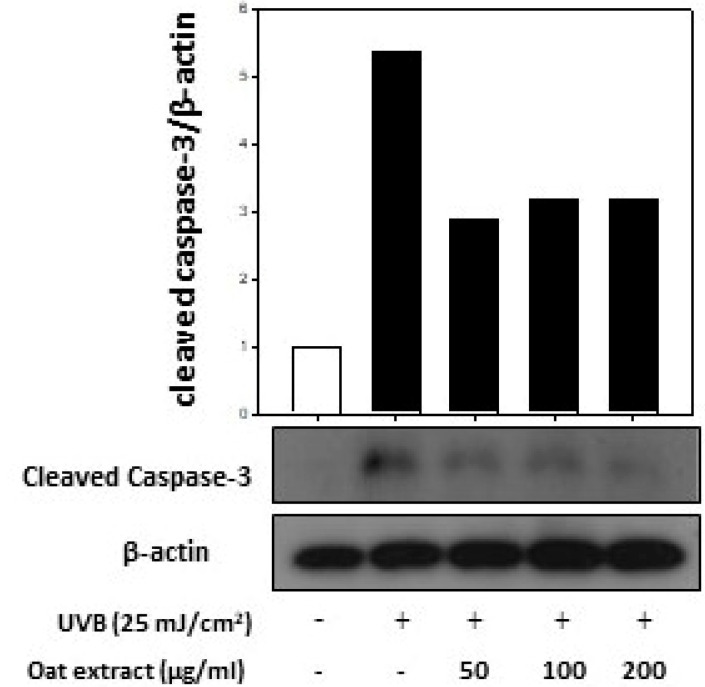
Effect of oat (*Avena sativa,* cv. Daeyang) 80% ethanol extract on cleaved caspase-3 protein level in human keratinocytes HaCaT. Cells were treated with various concentrations (50–200 μg/mL) of oat 80% ethanol extracts for 24 h, followed by incubation for 5 h after exposure of 25 mJ/cm^2^ UVB. Cleaved caspase-3 protein level was assessed using immunoblotting. β-Actin was used as an internal control.

In addition, the cleaved caspase-3 levels elevated by UVB exposure were also restored by the oat 80% ethanol extract treatment (Figure 7). These results indicate that the apoptosis induced by oxidative stress can be modified by antioxidant-rich oat extracts.

## 3. Materials and Methods

### 3.1. Preparation of Oat Extract

Daeyang and Choyang oat cultivars have been developed in the National Institute of Crop Science, Rural Development Administration (RDA), South Korea. Oat (*Avena sativa,* cv. Daeyang and Choyang) extracts were obtained from National Institute of Crop Science (Suwon, South Korea). Lyophilized oats (*Avena sativa,* cv. Daeyang and Choyang) were ground and extracted with water, 40% ethanol and 80% ethanol. The extracts were filtered through Whatman No. 2 paper (Whatman, Kent, UK), evaporated under vacuum condition (N-1200A, Eyela, Tokyo, Japan), and then freeze-dried (LP-10, Ilsin Biobase, Yangju, Korea). Oat (*Avena sativa*) extracts were kept at −80 °C until use. 

### 3.2. Reagents 

Lutein, zeaxanthin, HPLC grade water, acetonitrile, tetrahydrofuran, methanol, lutein and zeaxanthin were purchased from Sigma Aldrich (St Louis, MO, USA). Gamma-oryzanol was obtained from Wako (Osaka, Japan) and tocopherols and tocotrienols were purchased from Cayman Chemical (Ann Arbor, MI, USA). 2,7-Dicholorofluorescin diacetate (DCF-DA) kit and caspase-3 assay kit were purchased from Abcam (Cambridge, UK). Dulbecco’s Modified Eagle’s Medium (DMEM), Penicillin streptomycin, hydrogen peroxide, dimethyl sulfoxide (DMSO) and 3-(4,5-dimethylthiazol-2-y1)-2,5-diphenyltetrazolium bromide (MTT) were purchased from Sigma Aldrich (St. Louis, MO, USA). Fetal bovine serum (FBS) was obtained from Gibco (Waltham, MA, USA). RIPA buffer was purchased from Thermo Fisher Scientific (Waltham MA USA). Phosphate inhibitor and protease inhibitor were purchased from Gen DEPOT (Barker, TX, USA). The antibodies (caspase-3, -7, PARP, Bax, phospho-H2AX, phospho-p53, phospho-chk1, phospho-chk2 and β-actin) used in Western blot analysis were purchased from Cell Signaling Technology (Danvers, MA, USA).

### 3.3. Ultra-Performance Liquid Chromatography

Fat soluble micronutrients in oat extract were analyzed using a UPLC, as previously reported [48] with slight modification. The UPLC (ACQUITY, Waters Co., Milford, MA, USA) system was equipped with a BEH C18 column (1.7 μm, 2.1 mm × 50 mm, Waters Co., Milford, MA, USA), binary pump delivery system, autosampler and photodiode array detector. The mobile phase A was acetonitrile/methanol (7:3, *v*/*v*), and the mobile phase B was water. The gradient procedure was adapted as previously reported [48] with slight modification. Lutein (450 nm), zeaxanthin (450 nm), gamma-oryzanols (330 nm) and alpha-tocotrienol (292 nm) were quantified by each standard curve. Each peak was confirmed by retention time and its unique spectrum. The interassay coefficient of variation (CV) was under 4% (n = 10), and the intraassay CV was under 4% as well (n = 10).

### 3.4. DPPH Radical Scavenging Assay

The ability of oat to scavenge free radicals was determined by the DPPH radical scavenging activity, as previously reported [28]. Various concentrations of oat extract were dissolved in 80% ethanol, and then mixed with an equal volume of 0.2 mM DPPH solution. The mixtures were incubated at room temperature for 30 min in the dark, and absorbance was read at 517 nm (Molecular Device, Sunnyvale, CA, USA). Radical scavenging effect (%) = AD-AS/AD × 100, where AD is the absorbance of control and AS is the absorbance value of sample. 

### 3.5. Cell Culture

Human keratinocytes HaCaT cells were cultured in DMEM containing 10% FBS and antibiotics (100 units/mL penicillin, 100 μg/mL streptomycin and 250 ng/mL amphotericin B) under 5% CO_2_ at 37 °C with humidified air.

### 3.6. Cell Viability

MTT colorimetric assay was used to determine cell viability, as previously reported [28]. Briefly, HaCaT cells were seeded in 96-well plates and incubated overnight followed by pre-treatment with various concentrations (50–500 μg/mL) of oat (*Avena sativa*, cv. Daeyang) 80% ethanol extract for 24 h. Afterwards, medium containing oat 80% ethanol extract was removed and further incubated in the presence of H_2_O_2_ (50–500 μM) for 24 h. Five mg/mL of MTT solutions were added into the medium at a final concentration of 0.5 mg/mL and incubated for 3 h. After removing all medium, DMSO solution was added into each well to resuspend the MTT formazan. The absorbance was measured at 540 nm using a microplate reader (SpectraMax M2, Molecular Devices, Sunnyvale, CA, USA).

### 3.7. Intracellular Reactive Oxygen Species (ROS) Determination

To determine oxidative stress, intracellular ROS were assessed using a DCF-DA fluorescence assay [49]. Cells were grown in black well clear bottom 96-well plates for 24 h. After washing with phosphate buffered saline (PBS) two times, cells were stained with 25 μM of DCF-DA for 45 min in the dark. Then, the cells were treated with various concentrations (50–200 μg/mL) of oat (*Avena sativa*, cv. Daeyang) 80% ethanol extracts in the presence or absence of H_2_O_2_ (500 μM) for 3 h. The fluorescence was determined at 485 (excitation)/535 (emission) nm using microplate reader (Molecular Devices, Sunnyvale, CA, USA).

### 3.8. Immunoblotting

Cells were treated with various concentrations of oat (*Avena sativa*, cv. Daeyang) 80% ethanol extract for 24 h followed by 2 h of incubation with 500 μM of H_2_O_2_ or 5 h of incubation after exposure to 25 mJ/cm^2^ of UVB for sensor proteins of DNA damage and incubation with 500 μM of H_2_O_2_ for 24 h or incubation for 5 h after exposure to 25 mJ/cm^2^ of UVB for apoptotic genes. As previously reported [50], samples were lysed with an RIPA buffer containing 25 mM Tris-HCl at pH7.6, 150 mM NaCl, 1% NP-40, 1% sodium deoxycholate, 0.1% SDS and protease inhibitors. After cells were lysed, the supernatants were collected by centrifugation at 12,000 rpm for 10 min, and the protein content in the supernatant were normalized using the BCA protein assay (BioRad, Hercules, CA, USA). The equal amounts of protein were mixed with 4X sample buffer (250 mM of Tris-Cl at pH 6.8, 8% SDS, 40% glycerol, 8% β-mercaptoethanol, 0.01% bromophenol blue). Boiled samples were loaded into SDS-PAGE gels. After electrophoresis, the gel was transferred onto PVDF membrane (Millipore, Billerica, MA, USA). The membranes were blocked with 5% skim milk for 1 h at room temperature, and reacted with primary antibodies (cleaved caspase-3, cleaved caspase-7, phospho-H2AX, phospho-p53, cleaved PARP and actin) overnight at 4 °C. The PVDF membranes were then incubated with a horseradish peroxidase-conjugated secondary antibody for 1 h at room temperature. The protein band was developed using the enhanced chemiluminescence (GE Healthcare, Piscataway, NJ, USA). The intensity of the band signal was calculated using the Image J program provided by the NIH (https://imagej.nih.gov/ij/download.html) accessed on 20 May 2018.

### 3.9. Analysis of Caspase Enzyme Activity

Cells were treated with 200 μg/mL of oat (*Avena sativa*, cv. Daeyang) 80% ethanol extracts for 24 h, and further incubated with 500 μM H_2_O_2_ for 24 h. Caspases-3 activity was determined using colorimetric assay (Cayman Chemical, Ann Arbor, MI, USA), and all experiments were preformed according to the manufacturer’s instruction. Briefly, cells were pretreated with 200 μg/mL of oat (*Avena sativa*, cv. Daeyang) 80% ethanol extracts for 24 h, and further incubated with 500 μM of H_2_O_2_ for 24 h. After harvesting the cells, they were lysed with lysis buffer provided in the kit. After centrifuge, supernatant was reacted with 1 M DTT and 4 mM DEVD-p-NA substrate for 2 h at 37 °C. The absorbance was read at 405 nm using microplate reader (Molecular Devices, Sunnyvale, CA, USA).

### 3.10. Statistical Analysis

All experiments were performed in triplicate and expressed as mean ± SD. Data were analyzed using two-tailed unpaired Student’s *t*-test (Sigma plot 14) and considered as significant when *p* value was under 0.05.

## 4. Conclusions

In the current study, we found that oat extracts rich in antioxidants such as polyphenols, avenanthramides, γ-oryzanols, tocotrienols and carotenoids can protect the oxidative stress-induced cell damage through inhibiting the production of intracellular reactive oxygen species and blocking the phosphorylation of DNA damage-mediated genes such as H2AX, Chk1, Chk2 and p53, and consequently, reducing the activation of apoptosis-inducing genes such as caspases and PARP. Thus, this study suggests oat extract can be a good source of preventing skin damage from environmental stress.

## Data Availability

All the relevant data have been provided in the manuscript. The authors will provide additional details if required.

## Sample Availability

Samples of the compounds are not available from the authors.

## References

[1] Apel K., Hirt H. (2004). Reactive oxygen species: Metabolism, oxidative stress, and signal transduction. Annu. Rev. Plant Biol..

[2] Guo W., An Y., Jiang L., Geng C., Zhong L. (2009). The protective effects of hydroxytyrosol against UVB-induced DNA damage in HaCaT cells. Phytotherapy Res..

[3] Cross C.E., Halliwell B., Borish E.T., Pryor W.A., Ames B.N., Saul R.L., Mccord J.M., Harman D. (1987). Oxygen Radicals and Human Disease. Ann. Intern. Med..

[4] Aruoma O.I. (1998). Free radicals, oxidative stress, and antioxidants in human health and disease. J. Am. Oil Chem. Soc..

[5] Girsang E., Ginting C.N., Lister I.N.E., Gunawan K.Y., Widowati W. (2021). Anti-inflammatory and antiaging properties of chlorogenic acid on UV-induced fibroblast cell. PeerJ.

[6] Kanitakis J. (2002). Anatomy, histology and immunohistochemistry of normal human skin. Eur. J. Dermatol..

[7] Xiao T., Chen Y., Song C., Xu S., Lin S., Li M., Chen X., Gu H. (2021). Possible treatment for UVB-induced skin injury: Anti-inflammatory and cytoprotective role of metformin in UVB-irradiated keratinocytes. J. Dermatol. Sci..

[8] Nazir L.A., Tanveer M.A., Umar S.A., Love S., Divya G., Tasduq S.A. (2021). Inhibition of Ultraviolet-B Radiation Induced Photodamage by Trigonelline Through Modulation of Mitogen Activating Protein Kinases and Nuclear Factor-kappaB Signaling Axis in Skin. Photochem. Photobiol..

[9] Seo S.-H., Jeong G.-S. (2015). Fisetin inhibits TNF-α-induced inflammatory action and hydrogen peroxide-induced oxidative damage in human keratinocyte HaCaT cells through PI3K/AKT/Nrf-2-mediated heme oxygenase-1 expression. Int. Immunopharmacol..

[10] Nguyen C.N., Kim H.-E., Lee S.-G. (2013). Caffeoylserotonin Protects Human Keratinocyte HaCaT Cells against H2O2-Induced Oxidative Stress and Apoptosis through Upregulation of HO-1 Expression via Activation of the PI3K/Akt/Nrf2 Pathway. Phytotherapy Res..

[11] Bae S., Lee E.-J., Lee J.H., Park I.-C., Lee S.-J., Hahn H.J., Ahn K.J., An S., An I.-S., Cha H.J. (2013). Oridonin protects HaCaT keratinocytes against hydrogen peroxide-induced oxidative stress by altering microRNA expression. Int. J. Mol. Med..

[12] Tosh S.M., Bordenave N. (2020). Emerging science on benefits of whole grain oat and barley and their soluble dietary fibers for heart health, glycemic response, and gut microbiota. Nutr. Rev..

[13] El Khoury D., Cuda C., Luhovyy B.L., Anderson G.H. (2012). Beta glucan: Health benefits in obesity and metabolic syndrome. J. Nutr. Metab..

[14] Perrelli A., Goitre L., Salzano A.M., Moglia A., Scaloni A., Retta S.F. (2018). Biological Activities, Health Benefits, and Therapeutic Properties of Avenanthramides: From Skin Protection to Prevention and Treatment of Cerebrovascular Diseases. Oxidative Med. Cell. Longev..

[15] Handelman G.J., Cao G., Walter M.F., Nightingale Z.D., Paul G.L., Prior R.L., Blumberg J.B. (1999). Antioxidant capacity of oat (*Avena sativa* L.) extracts. 1. Inhibition of low-density lipoprotein oxidation and oxygen radical absorbance capacity. J. Agric. Food Chem..

[16] Van Hung P. (2016). Phenolic Compounds of Cereals and Their Antioxidant Capacity. Crit. Rev. Food Sci. Nutr..

[17] Hernandez-Hernandez O., Pereira-Caro G., Borges G., Crozier A., Olsson O. (2021). Characterization and antioxidant activity of avenanthramides from selected oat lines developed by mutagenesis technique. Food Chem..

[18] Lin T.-K., Zhong L., Santiago J.L. (2018). Anti-Inflammatory and Skin Barrier Repair Effects of Topical Application of Some Plant Oils. Int. J. Mol. Sci..

[19] Chon S.-H., Tannahill R., Yao X., Southall M.D., Pappas A. (2015). Keratinocyte differentiation and upregulation of ceramide synthesis induced by an oat lipid extract via the activation of PPAR pathways. Exp. Dermatol..

[20] Reich A., Estebaranz J.L., Bahadoran P., Guillot P., Carballido F., Aroman M.S. (2020). A spray containing extracts of oat plantlets and Uncaria tomentosa relieves pain associated with chronic inflammatory skin diseases and dermatological procedures. J. Eur. Acad. Dermatol. Venereol..

[21] Reynertson K.A., Garay M., Nebus J., Chon S., Kaur S., Mahmood K., Kizoulis M., Southall M.D. (2015). Anti-inflammatory activities of colloidal oatmeal (*Avena sativa*) contribute to the effectiveness of oats in treatment of itch associated with dry, irritated skin. J. Drugs Dermatol..

[22] Pazyar N., Yaghoobi R., Rafiee E., Mehrabian A., Feily A. (2014). Skin Wound Healing and Phytomedicine: A Review. Ski. Pharmacol. Physiol..

[23] Wollenberg A., Fölster-Holst R., Aroman M.S., Sampogna F., Vestergaard C. (2018). Effects of a protein-free oat plantlet extract on microinflammation and skin barrier function in atopic dermatitis patients. J. Eur. Acad. Dermatol. Venereol..

[24] Capone K., Kirchner F., Klein S.L., Tierney N.K. (2020). Effects of Colloidal Oatmeal Topical Atopic Dermatitis Cream on Skin Microbiome and Skin Barrier Properties. J. Drugs Dermatol..

[25] Juliano C., Cossu M., Alamanni M.C., Piu L. (2005). Antioxidant activity of gamma-oryzanol: Mechanism of action and its effect on oxidative stability of pharmaceutical oils. Int. J. Pharm..

[26] Minatel I.O., Han S.-I., Aldini G., Colzani M., Matthan N.R., Corrêa C.R., Fecchio D., Yeum K.-J. (2014). Fat-Soluble Bioactive Components in Colored Rice Varieties. J. Med. Food.

[27] Packer L., Weber S.U., Rimbach G. (2001). Molecular Aspects of α-Tocotrienol Antioxidant Action and Cell Signalling. J. Nutr..

[28] Yoon Y., Lee Y.-M., Song S., Lee Y.Y., Yeum K.-J. (2018). Black soybeans protect human keratinocytes from oxidative stress-induced cell death. Food Sci. Nutr..

[29] Hou Y., Peng S., Song Z., Bai F., Li X., Fang J. (2021). Oat polyphenol avenanthramide-2c confers protection from oxidative stress by regulating the Nrf2-ARE signaling pathway in PC12 cells. Arch. Biochem. Biophys..

[30] Purkait S., Bhattacharya A., Bag A., Chattopadhyay R.R. (2021). TLC bioautography–guided isolation of essential oil components of cinnamon and clove and assessment of their antimicrobial and antioxidant potential in combination. Environ. Sci. Pollut. Res..

[31] Liu R., Xu Y., Chang M., Liu R., Wang X. (2021). Interactions between alpha-tocopherol and gamma-oryzanol in oil-in-water emulsions. Food Chem..

[32] Liu R., Xu Y., Chang M., Tang L., Lu M., Liu R., Jin Q., Wang X. (2021). Antioxidant interaction of alpha-tocopherol, gamma-oryzanol and phytosterol in rice bran oil. Food Chem..

[33] Gholami F., Antonio J., Evans C., Cheraghi K., Rahmani L., Amirnezhad F. (2021). Tomato powder is more effective than lycopene to alleviate exercise-induced lipid peroxidation in well-trained male athletes: Randomized, double-blinded cross-over study. J. Int. Soc. Sports Nutr..

[34] Ge X., Jing L., Zhao K., Su C., Zhang B., Zhang Q., Han L., Yu X., Li W. (2021). The phenolic compounds profile, quantitative analysis and antioxidant activity of four naked barley grains with different color. Food Chem..

[35] Angeloni S., Spinozzi E., Maggi F., Sagratini G., Caprioli G., Borsetta G., Ak G., Sinan K., Zengin G., Arpini S. (2021). Phytochemical Profile and Biological Activities of Crude and Purified *Leonurus cardiaca* Extracts. Plants.

[36] Nowsheen S., Yang E. (2012). The intersection between DNA damage response and cell death pathways. Exp. Oncol..

[37] Harper J.W., Elledge S.J. (2007). The DNA Damage Response: Ten Years After. Mol. Cell.

[38] Patil M., Pabla N., Dong Z. (2013). Checkpoint kinase 1 in DNA damage response and cell cycle regulation. Cell. Mol. Life Sci..

[39] MacLaine N.J., Hupp T.R. (2011). How phosphorylation controls p53. Cell Cycle.

[40] Moiseeva O., Mallette F.A., Mukhopadhyay U.K., Moores A., Ferbeyre G. (2006). DNA damage signaling and p53-dependent senescence after prolonged beta-interferon stimulation. Mol. Biol. Cell.

[41] Pabla N., Huang S., Mi Q.S., Daniel R., Dong Z. (2008). ATR-Chk2 signaling in p53 activation and DNA damage response during cisplatin-induced apoptosis. J. Biol. Chem..

[42] Mah L.J., El-Osta A., Karagiannis T.C. (2010). gammaH2AX: A sensitive molecular marker of DNA damage and repair. Leukemia.

[43] Wang L., Je J.-G., Yang H.-W., Jeon Y.-J., Lee S. (2021). Dieckol, an Algae-Derived Phenolic Compound, Suppresses UVB-Induced Skin Damage in Human Dermal Fibroblasts and Its Underlying Mechanisms. Antioxidants.

[44] Li Y., Xia C., Yao G., Zhang X., Zhao J., Gao X., Yong J., Wang H. (2021). Protective effects of liquiritin on UVB-induced skin damage in SD rats. Int. Immunopharmacol..

[45] Hengartner M.O. (2000). The biochemistry of apoptosis. Nature.

[46] Cohen G.M. (1997). Caspases: The executioners of apoptosis. Biochem. J..

[47] Slee E.A., Adrain C., Martin S. (2001). Executioner Caspase-3, -6, and -7 Perform Distinct, Non-redundant Roles during the Demolition Phase of Apoptosis. J. Biol. Chem..

[48] Delpino-Rius A., Eras J., Marsol-Vall A., Vilaró F., Balcells M., Canela-Garayoa R. (2014). Ultra performance liquid chromatography analysis to study the changes in the carotenoid profile of commercial monovarietal fruit juices. J. Chromatogr. A.

[49] LeBel C.P., Ischiropoulos H., Bondy S.C. (1992). Evaluation of the probe 2′,7′-dichlorofluorescin as an indicator of reactive oxygen species formation and oxidative stress. Chem. Res. Toxicol..

[50] Lee Y.-M., Han S.-I., Won Y.-J., Lee E., Park E., Hwang S.-Y., Yeum K.-J. (2016). Black Rice with Giant Embryo Attenuates Obesity-Associated Metabolic Disorders in ob/ob Mice. J. Agric. Food Chem..

